# Exercise and colorectal cancer: a systematic review and meta-analysis of exercise safety, feasibility and effectiveness

**DOI:** 10.1186/s12966-020-01021-7

**Published:** 2020-09-24

**Authors:** Benjamin Singh, Sandra C. Hayes, Rosalind R. Spence, Megan L. Steele, Guillaume Y. Millet, Laurent Gergele

**Affiliations:** 1 Univ Lyon, UJM-Saint-Etienne, Inter-university Laboratory of Human Movement Biology, EA 7424, F-42023 Saint-Etienne, France; 2 Laboratoire Interuniversitaire de Biologie de la Motricité, Bâtiment IRMIS, 10 rue de la Marandière, 42270 Saint Priest en Jarez, France; 3 Griffith University, Menzies Health Institute Queensland, Brisbane, Queensland Australia; 4 Institute of Health and Biomedical Innovation, Queensland University of Technology, Musk Avenue, Kelvin Grove, Queensland Australia; 5 School of Clinical Medicine, University of Queensland, St. Lucia, Queensland Australia; 6 Institut Universitaire de France (IUF), Paris, France; 7 Hôpital Privé de la Loire, Saint-Étienne, France

**Keywords:** Colon, Colorectal, Rectal, Cancer, Neoplasm, Aerobic exercise, Resistance exercise, Exercise oncology

## Abstract

**Background:**

This meta-analysis evaluated the safety, feasibility and effect of exercise among individuals with colorectal cancer.

**Methods:**

A database search (CINAHL, Ebscohost, MEDLINE, Pubmed, ProQuest Health and Medical Complete, ProQuest Nursing, Science Direct) for randomised, controlled, exercise trials involving individuals with colorectal cancer, published before January 1, 2020 was undertaken. Safety (adverse events), feasibility (withdrawal and adherence rates) and effect data (health outcomes including quality of life, QoL) were abstracted. Risk difference (RD) and standardised mean differences (SMD) were calculated to compare safety and effects between exercise and usual care (UC). Subgroup analyses were conducted to assess whether outcomes differed by exercise mode, duration, supervision and treatment. Risk of bias was assessed using the Physiotherapy Evidence Database tool.

**Results:**

For the 19 trials included, there was no difference in adverse event risk between exercise and UC (RD = 0.00; 95% CI:–0.01, 0.01, *p* = 0.92). Median withdrawal rate was 12% (0–22%) and adherence was 86% (42–91%). Significant effects of exercise compared to UC were observed for QoL, fatigue, aerobic fitness, upper-body strength, depression, sleep and reduced body fat (SMD = 0.21–0.66, *p* < 0.05). Subgroup analyses suggested larger benefits (p < 0.05) for QoL and fatigue for supervised interventions; for QoL, aerobic fitness and reduced body fat for ≥12-week interventions; and for aerobic fitness when interventions were during chemotherapy.

**Conclusion:**

Although reporting of safety and compliance data was lacking in most trials, findings support that exercise is safe and feasible in colorectal cancer. Further, participation in mixed-mode exercise, including unsupervised exercise, leads to improvements in various health-related outcomes.

## Background

Colorectal cancer is the third most common cancer worldwide, with approximately 1.8 million new cases diagnosed in 2018 [1]. Median age at diagnosis is 69 and 66 years for females and males, respectively [2, 3], and over the past 20 years, improvements in screening, diagnosis and treatment have contributed to improvements in survival rates of up to 30% [4–6]. The 5-year relative survival rate currently sits at 65% [3], making colorectal cancer survivors the largest group of cancer survivors involving both females and males [7].

Treatment for colorectal cancer involves surgery, radiation therapy, chemotherapy, and/or targeted therapies (either alone or in combination). The majority (up to 98%) of patients diagnosed will receive surgery and at least one-third will receive chemotherapy and/or radiotherapy [8]. Surgery is associated with a high-risk of complication; approximately one-third of patients experience surgical treatment-related issues including wound complications, chest infections, anastomotic leakage and haemorrhaging [9]. Other common adverse effects associated with adjuvant treatments include pain, weakness, fatigue, diarrhea, cardiotoxicity, bowel dysfunction, anorectal dysfunction, sexual dysfunction, anxiety, depression, reduced physical fitness and function, and reduced quality of life (QoL, [10–13]). Impairments in social and role functioning, particularly the ability to participate in community activities, social activities, and undertake work and employment have also been reported by colorectal cancer survivors [14]. Further, more than 30% of patients will experience disease recurrence, contributing to threatened long-term survival [2]. Nonetheless, the number of individuals living with and beyond colorectal cancer is expected to continue to increase [15]. As such, there is a need for effective strategies to address common adverse treatment-related effects and improve the quality and duration of survivorship following colorectal cancer [16].

Observational findings indicate that physical activity is associated with higher overall, as well as disease-specific survival in individuals with colorectal cancer. Specifically, following colorectal cancer, higher post-diagnosis physical activity has shown to be protective against cancer-specific mortality (Hazard ratio, HR: 0.76 [95% CI: 0.58–0.99]) and all-cause mortality (HR: females: 0.62 [95% CI, 0.47–0.83]; males: 0.80 [95% CI, 0.74–0.87]) [17]. The effects of exercise following colorectal cancer on a limited number of survivorship outcomes (including fatigue, QoL and physical fitness) has been evaluated in three previous meta-analyses, each involving pooled analyses of 2 to 7 randomized controlled trials (RCTs), involving a total of 238 [18], 630 [19] and 628 participants who had completed treatment [20]. Findings showed no effect of exercise on fatigue (standardized mean difference, SMD = 0.08–0.21, [18–20] and QoL (SMD = 0.18–0.22, [18, 20]), and improvements in physical fitness (SMD = 0.59–0.72, [18, 20]). Due to the small number of studies contributing to the pooled analyses, as well as degree of heterogeneity among the included studies, the overall strength of review findings, and information about effect on various health outcomes across different phases of the cancer continuum was limited. Overall, previous systematic reviews and meta-analyses have focussed on evaluating the effects of exercise on health outcomes (e.g., QoL) post-treatment. To date, there has been no evaluation of the feasibility of exercise, and the potential risks associated with exercise pre-, during and following treatment (surgery and/or surgery plus adjuvant treatment) for colorectal cancer.

Over the past 10 years, there has been an exponential increase in the number of published exercise intervention trials among cancer populations, including individuals with colorectal cancer, providing opportunity to evaluate: 1) safety: the number, type and severity of adverse events; 2) feasibility: study recruitment, withdrawal and adherence rates; and 3) effect of exercise (as assessed immediately post-intervention) on colorectal survivorship outcomes including QoL, aerobic fitness and fatigue. As a secondary objective, we also explored the relationship between safety, feasibility, and effect, and intervention characteristics including exercise mode, degree of intervention supervision, intervention duration and timing with respect to treatment.

## Methods

### Search strategy and selection criteria

The protocol for this systematic review and meta-analysis was pre-registered on the PROSPRO registry (ID: CRD42020164152). The inclusion criteria were developed using the Participants, Intervention, Comparator, and Outcome (PICO) approach [21]. Participants: RCTs that involved participants with all stages of colorectal cancer, either preparing for, undergoing or having completed treatment were eligible. If a study involved participants with multiple cancer types (in addition to colorectal cancer), the study was eligible if results for the participants with colorectal cancer were reported separately. Intervention: RCTs that evaluated exercise interventions were eligible. Exercise was considered to be any form of planned, structured, and repetitive bodily movement undertaken to improve or maintain fitness, performance or health [22]. An RCT evaluating an exercise intervention was considered as a comparative study designed to test the safety, feasibility, or efficacy of an exercise intervention with random allocation of participants to groups. Subgroups for exercise mode were aerobic, resistance, combined (mixed-mode) or other. Any form of exercise that was not aerobic or resistance was considered ‘other exercise’ (e.g., yoga). Studies were eligible regardless of supervision, type of intervention delivery, intervention duration or exercise dose. Interventions conducted at any time pre-, during or following treatment were eligible. Studies that involved exercise in addition to other interventions were excluded if the results of exercise could not be isolated. Comparators: To be eligible, studies needed to include a usual care group.

An electronic database search was undertaken using combinations of MeSH and free-text words for “colorectal” and “physical activity” (see Supplementary content 1 for the full search details for all databases). The following databases were searched: Allied Health Source, CINAHL, Cochrane, Ebscohost, PubMed, ProQuest Health and Medical Complete, ProQuest Nursing, MEDLINE, Science Direct and SPORTDiscus. Database searches were limited to peer-reviewed journal articles published in English-language prior to January 1, 2020.

### Outcomes of interest

#### Safety and feasibility

We evaluated exercise safety by assessing adverse events, defined as any undesirable health- or medical-related event that occurred during the trial. Adverse events were grouped as either non-exercise adverse events (events reported in the paper as unrelated to exercise) or exercise-related adverse events (events which were reported to occur during, or as a direct result of exercise) [23]. We categorised adverse events based on severity using the Common Terminology Criteria for Adverse Event (Version 5.0) as either grade 1 (asymptomatic or mild symptoms, clinical or diagnostic observations only and/or intervention not indicated); grade 2 (moderate, minimal, local or non-invasive intervention required and/or limiting age-appropriate activities of daily living); grade 3 (severe or medically significant but not immediately life-threatening, hospitalisation and/or prolongation of hospitalisation indicated, disabling and limiting self-care activities of daily living); grade 4 (life-threatening consequences and urgent intervention indicated), or; grade 5 (death). To minimise under-reporting of adverse events, we considered any withdrawal that occurred due to health, medical or disease-related reasons as an adverse event (e.g., illness or cancer recurrence) [23]. Withdrawals that occurred due to non-health-related reasons (e.g., time/travel constraints) were not considered adverse events. If the severity of an adverse event was not reported and the event resulted in study withdrawal, or if a participant withdrew from a trial due to health, medical or disease-related reasons, these events were categorised as grade 3 [23]. If a study did not report on the occurrence of adverse events, and no health, medical or disease-related withdrawals occurred, it was considered that no adverse events had occurred.

Recruitment, withdrawal (plus reasons for withdrawals) and exercise adherence rates were assessed to determine feasibility. Recruitment rates were calculated as the percentage of individuals who were eligible and consented to participate in the study. Withdrawal rates were computed as the percentage of participants who commenced but did not complete the study. Adherence rates were calculated as a percentage of scheduled exercise sessions that were completed.

#### Health outcomes

Meta-analyses (including subgroup analyses) were undertaken on health-related outcomes that were assessed and reported in at least 2 studies. These were objectively-assessed and/or self-reported QoL, aerobic fitness, fatigue, upper-body strength, lower-body strength, anxiety, depression, sleep, body fat percentage and body mass index.

### Data extraction

One investigator (BS) screened the titles and abstracts of all records that were identified during the electronic database search. The reference lists of relevant articles (original studies and reviews) were also screened manually to identify potentially eligible studies. The full text of articles that were deemed potentially eligible based on the title or abstract were then retrieved and screened to assess eligibility. Data extracted from each study into tabular format by one investigator (BS) included study and participant characteristics, intervention features, adverse events, recruitment details, exercise adherence and outcomes assessed.

The Physiotherapy Evidence Database (PEDro) tool was used to assess the quality of included RCTs by two investigators (BS and MS), and discrepancies (*n* = 26) were resolved with discussion with a third investigator (SH). The PEDro tool is a valid and reliable tool for assessing risk of bias in RCTs [24, 25] and consists of 11 items (eligibility criteria, random allocation, allocation concealment, baseline differences between groups, subject blinding, therapist blinding, assessor blinding, attrition, intention-to-treat analyses, between-group statistical comparisons and reporting of measures). RCTs with a score of 6 or greater (of a possible 10 points) were graded as high quality, and trials receiving less than 6 were graded as low quality [25, 26].

### Statistical analyses

#### Meta-analysis of adverse events

Adverse events were analysed as a count variable. A Mantel-Haenszel random effects model was used to pool and compare the total number of adverse events in exercise versus usual care groups. For the meta-analysis of adverse events, the risk difference (RD) and 95% confidence intervals were calculated, with a positive value for RD favouring the safety of exercise (i.e., indicating a lower adverse event risk with exercise compared with usual care). The RD was used as the effect measure to ensure that studies that reported zero adverse events (i.e., no difference between exercise and usual care) would not be excluded from the meta-analysis [27, 28]. Sensitivity analyses, excluding studies that did not report whether adverse events were measured were also conducted [23]. Due to minimal contact with research staff, grade 1–2 events were likely to have been less-comprehensively evaluated and reported for those in the usual care groups. Additionally, grade 1–2 events were likely to include normal physiological responses to exercise (e.g., mild muscle soreness), rather than potentially avoidable adverse events. Therefore, while all adverse events (grade 1–5) were reported using descriptive statistics, only adverse events that were grade 3 or higher were included in the meta-analysis.

#### Feasibility

Due to non-normally distributed data, overall recruitment, withdrawal and exercise adherence rates were reported using median, minimums and maximums. Cut-off values for the feasibility criterion (a recruitment rate of ≥25% [29]; a withdrawal rate of < 25% [30]; and adherence of ≥75% [30]) were established a priori as clinically relevant based on previous studies.

#### Meta-analysis of health outcomes

All health outcomes of interest were analysed as continuous variables and involved comparisons of post-intervention means and standard deviations (SDs) between exercise and usual care groups. Meta-analyses were conducted using standardised mean differences (SMDs) to allow comparison of data from different scales using RevMan software (version 5.4). R statistical software (version 4.0.2) was used to create forest plots. When means and SDs were not reported in a study (*n* = 2 studies), authors were contacted (*n* = 0 responded), or calculated based on reported data using recommended formulas (e.g., using sample size, median and range) [31]. If more than one method of assessing an outcome was used in a study, the method considered as being the gold standard or the method with demonstrated reliability and validity was used.

For each meta-analysis, data were combined at the study level. We assessed publication bias by plotting RDs or SMDs against corresponding standard errors and evaluating asymmetries or missing sections within the funnel plot [32]. Cochran’s Q test and the *I*^2^ statistic were used to assess statistical heterogeneity to quantify the proportion of the overall outcome attributed to variability [33, 34]. The following values were used to assess the amount of heterogeneity: *I*^2^ = 0 to 29%, no heterogeneity; *I*^2^ = 30 to 49%, moderate heterogeneity; *I*^2^ = 50 to 74%, substantial heterogeneity; and *I*^2^ = 75 to 100%, considerable heterogeneity [34]. Planned subgroup analyses were undertaken to evaluate the effects of: 1) exercise mode (aerobic, resistance, combined and ‘other’); 2) supervision (supervised [at least half of the exercise sessions involved face-to-face supervision], and unsupervised [less than half of the exercise sessions involved face-to-face supervision]); 3) duration (12 weeks or less and more than 12 weeks), and 4) timing of the intervention in relation to surgery and chemotherapy (pre-treatment [surgery], during treatment [chemotherapy] or post-treatment [surgery and/or chemotherapy]). Sensitivity analyses were also undertaken by evaluating high-quality studies only (based on a PEDro score of 6 or higher) on adverse events, recruitment, withdrawal and adherence rates, and health outcomes. Standardised classifications for the magnitude of effect were used (0.20 = small effect; 0.20–0.50 = medium effect; and greater than 0.50 = large effect [35]. A *p*-value of < 0.05 was considered statistically significant.

## Results

### Literature search

Following a search of databases, 1264 articles were identified (Supplementary content 2). After removal of duplicates and screening of titles and abstracts, 125 publications were retrieved and examined. Of these, 106 were excluded and 19 trials were included in the review (low quality, *n* = 2 [11%]; high quality, *n* = 17 [89%], Supplementary content 3). Two trials involved two exercise intervention arms in addition to a usual care group; these included evaluation of a high- versus low-dose exercise intervention [36] and evaluation of a high-intensity supervised intervention versus a low-moderate intensity unsupervised intervention [37]. Therefore, data from a total of 21 exercise intervention arms and 19 usual care arms were included.

### Study and participant characteristics

Median sample size was 39 (range: 18–284) and participant mean age was 64.6 years (SD = 6.9). Time since diagnosis was reported in 10 trials (53%) and ranged between 8.8 and 44.8 months (median = 18.4). Most trials involved participants with colorectal cancer (*n* = 13, 68%, [38–50]) and six trials (*n* = 6, 32%, [36, 37, 51–54]). Only one trial (5%, [47]) included patients with metastatic (stage IV) disease. Four trials (21%) evaluated the role of exercise pre-surgery [38, 40, 42, 44], five (26%) were during chemotherapy [37, 41, 43, 47, 54] and 10 (53%) were post-treatment [36, 39, 45, 46, 48–53].

### Intervention characteristics

Details of intervention characteristics are shown in Table 1. Intervention durations ranged between 1 week [51] and 6 months [36] (median 12 weeks). Four studies (*n* = 4, 21%) evaluated aerobic exercise only [36, 41, 44, 49], 14 studies (74%) evaluated mixed-mode exercise [37–40, 42, 43, 45–48, 51–54], one study (*n* = 1, 5%, [50]) evaluated other modes of exercise (yoga) and no studies evaluated resistance exercise alone. Home-based, unsupervised exercise was prescribed for 10 (53%) of the interventions [36, 40–42, 44–46, 48, 49, 53]. Eight trials (*n* = 8 [42%]) involved supervised interventions (i.e., over half of the exercise sessions involved face-to-face supervision [38, 39, 43, 47, 50–52, 54], and one trial (*n* = 1, 5%) involved a supervised and an unsupervised intervention group [37]. Supervised interventions were conducted at a range of facilities including hospital, clinical, university or rehabilitation settings. Supervision in these trials was provided by a physical therapist/physiotherapist (*n* = 5, [37, 39, 47, 51, 54]), an exercise physiologist (*n* = 2, [38, 52]), a cardiac physiotherapist/nurse (*n* = 1, [43]), and a yoga instructor (n = 1, [50]).

**Table 1:** Exercise details of included studies characterised by type, frequency, intensity, exercise duration and intervention length

	Pre-treatment (surgery)	During (chemotherapy) and post-treatment (surgery and/or chemotherapy)
**Type**	Aerobic: recumbent stepper, treadmill, outdoor walking, cycling or jogging (according to patient abilities and preference). Resistance: resistance training consisted of 6–10 exercises targeting major muscle groups of the upper- and lower-body; Theraband exercises.	Aerobic: brisk walking, jogging, running (treadmill), rowing ergometers, cycling ergometers, recumbent stepper. Resistance: Free weights, machine exercises and Therabands for major muscle groups (arms, abdominal muscles, thigh, and gluteus region; all major muscles of the upper- and lower-body). Other: Yoga.
**Frequency**	Supervised: 1 session per week. Unsupervised: 3–7 sessions per week.	Supervised sessions: 1–5 supervised sessions per week (including group-based and one-on-one supervised sessions). Unsupervised sessions: 1–7 sessions per week. In-hospital patients: daily supervised exercise.
**Intensity**	Aerobic: 60–70% of HRmax; 50% of age-predicted HRmax; 40% of HRR (starting intensity); 12–20 RPE (6–20 Borg scale). Resistance: 8–15 repetitions, 1–2 sets per exercise, dependent on volitional fatigue; 12 RPE (6–20 Borg scale).	Aerobic: continuous and interval bouts at 50–95% HRmax; 11–15 RPE (6–20 Borg scale); 40–80% HRR. Resistance: 8–15 repetitions, 1–3 sets per exercise (2–8 exercises targeting major muscle groups); 6–10 RPE (0–10 scale); 12–14 RPE (Borg 6–20 scale); 60–80% of one-repetition maximum.
**Duration**	20–60 min sessions (including warm-up and stretching).	15–90 min sessions (including warm-up and stretching).
**Length**	4 weeks.	7-days to 6-months.

*HRmax* maximal heart rate, *HRR* heart rate reserve, *RPE* rating of perceived exertion

### Safety - summary of adverse events

Of the 19 studies included in this review, four (*n* = 4, 21%) explicitly reported that no adverse events had occurred, while 9 (47%) made no mention of adverse events (i.e., whether adverse events occurred or not). Of the four studies that reported no adverse events, two of these reported the occurrence of participant withdrawals due to health or medical-related reasons.

#### Adverse events in exercise participants

There were 160 adverse events among 670 participants allocated to exercise (grade 1: *n* = 127 events; grade 2: *n* = 14 events; grade 3: n = 12 events; grade 4: *n* = 5 events; grade 5: *n* = 2 events; Table 2). The most common adverse events among exercise participants were low-severity muscle pain, stiffness or soreness (*n* = 65 events, grade 1) and fatigue (*n* = 7 events, grade 1). Of the 160 reported events, the 7 (4%) exercise-related adverse events were neck and abdominal discomfort during exercise (which limited exercise ability; n = 6 events, grade 2) and hip pain following exercise (*n* = 1 event, grade 1).

**Table 2:** Adverse events by grade of severity described for those in the exercise and usual care groups

AE grade	Exercise (160 adverse events, 670 participants) Total number of adverse events^a^ - exercise-related adverse events		Usual care (98 adverse events, 623 participants) Total number of adverse events^a^ - exercise-related adverse events	
1	Grade 1 adverse events: 127–1		Grade 1 adverse events: 73–0	
	Low-severity musculoskeletal symptoms (pain, stiffness, soreness, tendonitis, cramp or arthritis) (65–1) Fatigue (7–0) Flu-like symptoms (6–0) Palpitations (5–0) Bruising (5–0) Dizziness/vertigo (5–0) Headache (4–0) Diarrhea (3–0) Dry mouth (3–0) Toothache (3–0)	Otitis media (3–0) Fall (3–0) Sinus pain (3–0) External ear inflammation (2–0) Shortness of breath (2–0) Neuralgia (2–0) Mouth pain (1–0) Non-cardiac chest pain (1–0) Upper-respiratory infection (1–0) Constipation (1–0) Vomiting (1–0) Lymph node pain (1–0)	Low-severity musculoskeletal symptoms (pain, stiffness, soreness, tendonitis, cramp or arthritis) (41–0) Fatigue (10–0) Shortness of breath (2–0) Diarrhea (2–0) Flu-like symptoms (2–0) Bruising (2–0) Dizziness (2–0) Dry eye (1–0) Dry mouth (1–0)	Palpitations (1–0) Edema in limbs (1–0) Non-cardiac chest pain (1–0) Fall (1–0) Arthritis (1–0) Headache (1–0) Neuralgia (1–0) Sinus pain (1–0) Vomiting (1–0) Chills (1–0)
2	Grade 2 adverse events: 14–6		Grade 2 adverse events: 4–0	
	Musculoskeletal pain (6–6) Chest pain (2–0) Wound infection at the site of surgery (1–0) Blurred vision (1–0)	Gastroesophageal reflux disease (1–0) Edema limbs (1–0) Hyperglycaemia (1–0) Peripheral neuropathy (preventing exercise) (1–0)	Postoperative ileus (1–0) Hyperthyroidism (1–0) Gastroesophageal reflux disease (1–0) Chest pain (1–0)	
3^b^	Grade 3 adverse events: 12–0		Grade 3 adverse events: 14–0	
	Unspecified medical problem leading to withdrawal (5–0) Postoperative ventral hernias (2–0)	Fatigue and malaise (led to withdrawal) (2–0) Stroke (2–0) Acute illness (1–0)	Unspecified medical reasons (6–0) Mental reasons/depression (3–0) Postoperative ventral hernias (1–0) Microhaematuria (1–0)	Acute illness (1–0) Progressive disease with cough (1–0) Psychological reasons leading to withdrawal (1–0)
4	Grade 4 adverse events: 5–0		Grade 4 adverse events: 5–0	
	Cancer recurrence (1–0) Emergency surgery required (1–0) Pneumonia (1–0)	Metastasis (1–0) Diagnosed with lung cancer (1–0)	Metastasis (3–0) Cancer recurrence (2–0)	
5	Grade 5 adverse events: 2–0		Grade 5 adverse events: 2–0	
	Death (2–0; n = 1 due to perforated cholecystitis; *n* = 1 unspecified)		Death (2–0; n = 1 was due to sepsis; n = 1 unspecified)	

*AE* adverse events

Adverse events were classified using the Common Terminology Criteria as: grade 1- asymptomatic or mild symptoms; grade 2- moderate, minimal, local or non-invasive intervention indicated and limiting age-appropriate instrumental activities of daily living; grade 3- severe or medically significant but not immediately life-threatening; grade 4- life-threatening consequences and urgent intervention indicated, or; grade 5- death

^a^ Includes all adverse events (both exercise- and non-exercise related)

^b^Adverse events in which the severity was not reported were considered Grade 3 or higher if the event led to study withdrawal

#### Adverse events in usual care participants

For participants allocated to usual care (*n* = 623), 98 adverse events were reported (grade 1: *n* = 73 events; grade 2: *n* = 4 events; grade 3: *n* = 14 events; grade 4: *n* = 5; grade 5: *n* = 2, Table 2). The most common adverse events among usual care participants were low-severity musculoskeletal symptoms (pain, stiffness, soreness, tendonitis, cramp, arthritis; *n* = 41 events, grade 1) and fatigue (*n* = 10 events, grade 1).

#### Meta-analyses of adverse events

**Fig. 1:**
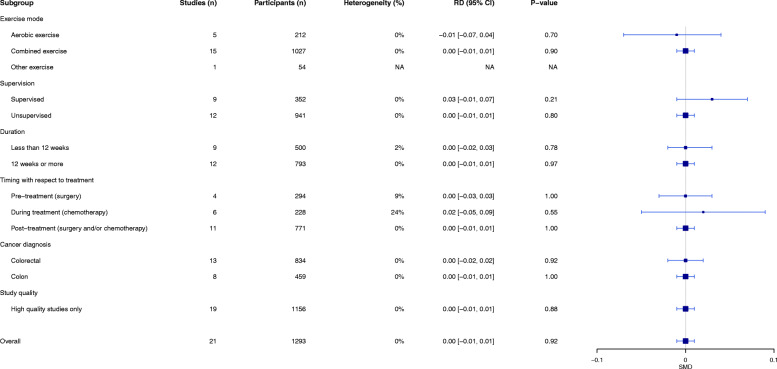
Meta-analysis results of all grade 3 to 5 adverse events in exercise compared to usual care presented as overall with subgroup analyses for 1) exercise mode, 2) supervision, 3) intervention length, 4) timing with respect to treatment, 5) cancer diagnosis, and 7) high quality studies only (n = 19 trials). Notes: Overall equals n = 21 because n = 2 studies involved multiple intervention arms; positive scores for risk difference indicate a lower risk of an adverse event with exercise compared to usual care

Pooled analyses of 19 RCTs (21 exercise arms) involving 1293 participants (exercise: *n* = 670; usual care: *n* = 623) showed no difference in the risk of a grade 3–5 adverse event between exercise and usual care (*n* = 40 adverse events [exercise: *n* = 19 events; usual care: *n* = 21 events], RD: 0.00 [95% CI = − 0.01, 0.01]; *p* = 0.92; *I*^2^ = 0%, Fig. 1). As shown in Fig. 1, subgroup analyses indicated that results were similar irrespective of intervention, treatment and cancer characteristics. Additionally, sensitivity analyses shows the results remain unchanged when restricted to only those studies which specifically mentioned the presence or absence of adverse events (RD: 0.00 [95% CI = − 0.03, 0.03]; *p* = 0.86, *I*^2^ = 0%), as well as when restricted to only studies of high quality (RD: 0.00 [95% CI: − 0.01, 0.01]; *p* = 0.88, *I*^2^ = 0%).

#### Feasibility outcomes: recruitment, withdrawals, and exercise adherence

Recruitment, withdrawal and adherence rates are shown in Table 3, with all rates meeting our a priori acceptability criteria. *Recruitment rates:* Study recruitment rates were based on data from 16 studies (*n* = 3 studies did not report recruitment rate) and were calculated to have an overall rate of 38% (range: 4–91%). *Withdrawals:* Median withdrawal rate was 12% (range: 0–22%), across a total of 21 intervention groups (*n* = 19 trials). By comparison, withdrawal rate was 11% (range: 0–31%) in the usual care groups. There was a total of 89 withdrawals (out of 670 participants) from the intervention groups (*n* = 18 [20%] due to health-related reasons; *n* = 71 [80%] due to non-health-related reasons) and 85 (out of 623 participants) withdrawals from usual care groups (*n* = 16 [19%] due to health-related reasons; *n* = 69 [81%] due to non-health-related reasons, Supplementary content 4). Unspecified non-health (i.e., non-medical) reasons were the most common reason for withdrawal in both groups (exercise: *n* = 32 withdrawals; usual care: *n* = 29 withdrawals; see Supplementary content 4 for all reasons for withdrawals). *Exercise adherence:* Overall median adherence to the scheduled number of exercise sessions was 86% (range: 42–98%, Table 3).

**Table 3:** Study recruitment rates, withdrawal rates and exercise adherence rates reported as overall, and for exercise mode, intervention supervision, intervention length, timing with respect to treatment, cancer diagnosis and study quality

	Recruitment rate (%) Median (minimum, maximum) [*n* = 16 trials]	Withdrawal rate (%)^a^ Median (minimum, maximum)		Adherence rate (%) Median (minimum, maximum) [n = 14 groups^a^ from *n* = 12 trials]
		Exercise [n = 21 groups from 19 trials^a^]	Usual care [*n* = 19 groups from 19 trials]	
Overall	38 (4–91)	12 (0, 22)	11 (0, 31)	86 (42, 98)
Mode				
Aerobic exercise	28 (15, 38)	5 (0, 14)	8 (0, 8)	76 (42, 93)
Mixed mode exercise	41 (4, 91)	12 (0, 90)	15 (0, 31)	88 (61, 98)
Other exercise	−^b^	− ^b^	− ^b^	− ^b^
Supervision				
Supervised	39 (10, 91)	11 (0, 22)	18 (0, 31)	88 (53, 98)
Unsupervised	37 (4, 84)	12 (0, 21)	8 (0, 21)	76 (42, 93)
Length				
< 12 weeks	61 (18, 91)	12 (0, 22)	18 (0, 31)	85 (53, 98)
≥ 12 weeks	33 (4, 54)	12 (0, 21)	8 (0, 21)	89 (42, 93)
Treatment				
Pre-treatment	88 (84, 91)	14 (0, 16)	11 (0, 18)	78 (74, 98)
During treatment	33 (26, 39)	12 (5, 14)	8 (0, 31)	75 (42, 89)
Post-treatment	28 (4, 61)	11 (0, 22)	13 (0, 21)	88 (53, 93)
Cancer diagnosis				
Colorectal	27 (4, 91)	11 (0, 22)	14 (0, 31)	77 (42, 98)
Colon only	39 (10, 61)	12 (0, 14)	8 (0, 19)	89 (61, 93)
Rectal only	−^b^	−^b^	−^b^	−^b^
Study quality				
Low	−^b^	−^b^	-^b^	-^b^
High	38 (4, 91)	12 (0, 22)	11 (0, 31)	88 (42, 98)

^a^ Reported by intervention groups because n = 2 trials involved multiple intervention groups

^b^ Insufficient number of studies

#### Health outcomes: assessment of outcomes

There was a sufficient number of studies to allow for the conduct of meta-analyses on 10 health outcomes including QoL, aerobic fitness, fatigue, upper-body strength, lower-body strength, sleep, anxiety, depression, body fat and body mass index (Supplementary content 5 presents an overview of instruments and methods used to assess these outcomes).

#### Meta-analyses results: exercise versus usual care

**Fig. 2:**
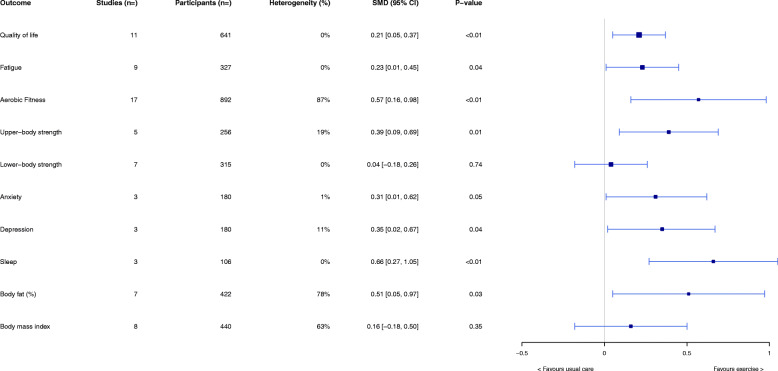
Meta-analysis results of all health outcomes presented as overall effects. SMD: standardised mean difference (positive scores favour exercise; negative scores favour usual care)

An overview of the main effects for all health outcomes is shown in Fig. 2. Compared with usual care, there were medium to large effects supported statistically (SMDs range: 0.21–0.66; all *p* < 0.05) in favour of exercise for all health outcomes of interest, with the exception of lower-body strength, anxiety and body mass index (SMD range: 0.04–0.31; all *p* ≥ 0.05). While results following sensitivity analyses (restricting analyses to data from studies rated as high quality) showed that effect sizes were attenuated for aerobic fitness (SMD: 0.39, 95% CI: 0.12–0.65; *p* < 0.01 [high-quality studies only] vs SMD: 0.59, 95% CI: 0.16, 0.98; p < 0.01 [all studies]), they continued to be supported statistically. Further, effects size remained relatively unchanged for all other outcomes following sensitivity analyses.

#### Subgroup effects

Findings from exploratory, subgroup analyses suggest benefit through exercise interventions, irrespective of mode, level of supervision, duration or timing with respect to chemotherapy or surgery for the majority of health outcomes. However, compared with effect size reported following the primary analyses, effect sizes were larger for specific outcomes under specific intervention conditions (Table 4). These include: for QoL and fatigue when interventions were supervised; for aerobic fitness, upper-body strength and reduced body fat when interventions were unsupervised; for QoL, aerobic fitness and reduced body fat when interventions were ≥ 12 weeks in duration; for QoL and reduced body fat when interventions were conducted post-treatment; for aerobic fitness when interventions were during chemotherapy; and for aerobic fitness and upper-body strength in trials that involved combined colorectal patients (Table 4).

**Table 4:** Summary of outcomes that had statistically significant effects based on subgroups with two or more studies

Health outcomes	No. of studies	Total participants		I^2^ (%)	Standardized mean difference (95% CI)	p-value
		Exercise	Usual care			
Quality of life						
Exercise mode						
Aerobic exercise	4	108	83	0	0.06 (−0.23, 0.35)	0.69
Mixed mode exercise	6	202	194	0	0.25 (0.05, 0.44)	0.02*
Other	1	−^a^	−^a^	−^a^	−^a^	−^a^
Supervision						
Supervised	5	77	73	0	0.39 (0.07, 0.72)	0.02*
Unsupervised	6	260	231	0	0.15 (−0.03, 0.33)	0.10
Intervention duration						
< 12 weeks	2	44	40	0	0.31 (−0.10, 0.98)	0.12
≥ 12 weeks	9	293	264	0	0.19 (0.02, 0.36)	0.02*
Treatment						
Pre-treatment	0	−^a^	−^a^	−^a^	−^a^	−^a^
During treatment	5	111	76	0	0.05 (−0.24, 0.35)	0.73
Post-treatment	6	226	228	0	0.27 (0.08, 0.45)	< 0.01*
Fatigue						
Supervision						
Supervised	4	59	61	0	0.43 (0.06, 0.79)	0.02*
Unsupervised	5	116	91	0	0.12 (−0.17, 0.40)	0.42
Aerobic fitness						
Exercise mode						
Aerobic exercise	5	123	100	0	0.46 (0.18, 0.73)	< 0.01*
Mixed mode exercise	12	339	330	91	0.64 (0.07, 1.20)	0.01*
Other exercise	0	−^a^	−^a^	−^a^	−^a^	−^a^
Supervision						
Supervised	7	136	129	80	0.45 (−0.14, 1.03)	0.14
Unsupervised	10	326	301	91	0.65 (0.08, 1.21)	0.03*
Intervention duration						
< 12 weeks	7	209	206	94	0.47 (−0.43, 1.38)	0.31
≥ 12 weeks	10	253	224	56	0.56 (0.25, 0.87)	< 0.01*
Timing regarding treatment						
Pre-surgery	3	136	137	98	1.00 (−0.72, 2.72)	0.25
During chemotherapy	5	115	81	46	0.72 (0.27, 1.17)	< 0.01*
Post-treatment	9	211	212	68	0.28 (−0.09, 0.0.65)	0.13
Cancer						
Colorectal cancer	10	371	338	91	0.55 (0.01, 1.08)	0.04*
Colon only	7	91	92	78	0.61 (−0.07, 1.28)	0.08
Rectal only	0	−^a^	−^a^	−^a^	−^a^	−^a^
Upper-body strength						
Exercise mode						
Aerobic exercise	0	−^a^	−^a^	− ^a^	−^a^	− ^a^
Mixed mode exercise	5	132	124	19	0.39 (0.09, 0.69)	0.01*
Other exercise	0	−^a^	−^a^	−^a^	−^a^	− ^a^
Supervision						
Supervised	2	24	21	0	0.10 (−0.49, 0.69)	0.75
Unsupervised	3	108	103	30	0.47 (0.11, 0.83)	0.01*
Cancer						
Colorectal cancer	3	117	108	36	0.43 (0.07, 0.78)	0.02*
Colon only	2	15	16	12	0.14 (−0.62, 0.90)	0.72
Rectal only	0	−^a^	−^a^	−^a^	−^a^	−^a^
Body fat						
Supervision						
Supervised	2	39	42	21	0.20 (−0.29, 0.69)	0.43
Unsupervised	5	154	187	85	0.67 (0.05, 1.30)	0.03*
Intervention duration						
< 12 weeks	2	39	42	21	0.20 (−0.29, 0.69)	0.43
≥ 12 weeks	5	154	187	85	0.67 (0.05, 1.30)	0.03*
Treatment						
Pre-treatment	0	−^a^	−^a^	−^a^	−^a^	−^a^
During treatment	1	−^a^	−^a^	− ^a^	−^a^	− ^a^
Post-treatment	6	162	167	80	0.63 (0.09, 1.18)	0.02*

^a^ Insufficient number of studies

* Statistically significant (*p* < 0.05)

## Discussion

Findings from this meta-analysis suggest that exercise is safe and feasible for individuals with colorectal cancer during and following treatment. There is also evidence to support that exercise is effective for improving QoL, fitness, fatigue, upper-body strength, sleep, depression, and body fat following diagnosis of colorectal cancer.

Participation in exercise was associated with a low adverse event risk, occurring in approximately 1% of participants, and there was no difference observed in grade III or higher adverse events between 1293 exercise and usual care participants (19 RCTs, 21 exercise arms). Of the grade I and II adverse events reported by those in the exercise group, most were low-grade (i.e., 79% were grade I, and no exercise-related events were grade 3 or higher) and reflected common responses to exercise (e.g., muscle soreness). Previous systematic reviews have either reported no adverse events [55], or not commented on adverse events in colorectal patients [18, 19]. However, previous reviews have been more limited in the ability to report on safety due to the low number of studies included, as well as poorer reporting of adverse events by early original research included in these reviews. Moreover, past reviews have focussed only on the post-treatment timepoint (i.e., not pre- or during treatment). While improvements in the assessment and reporting of adverse events has improved over the past decade, our low risk of adverse events finding should still be interpreted with caution. Almost one-half of included studies (*n* = 9, 47%) made no mention of adverse events (i.e., whether they occurred or not). Moreover, in the studies that reported adverse events, most did not comprehensively describe adverse event monitoring and recording procedures. Nonetheless, our subgroup findings provide some confidence that exercise safety can be maintained even when conducted during chemotherapy, under unsupervised conditions and including resistance exercise.

The recruitment rate (38%), low withdrawal rate (12%) and high exercise adherence rate (86%) reported here are consistent with those previously reported in exercise interventions among other cancer types, including breast cancer [23]. This supports the feasibility of exercise including for those with colorectal cancer. However, low withdrawals and high adherence may also be reflective of a recruitment bias, whereby those individuals who agree to participate in exercise trials may have higher exercise readiness compared with those who are less likely to volunteer [56]. Further, approximately one-third (*n* = 6, 32%) of studies included in this review excluded participants with metastatic (stage IV) disease and presence of comorbidities and only one trial specifically involved participants with metastatic disease. Given that one-quarter of individuals are diagnosed with metastatic disease [57] and one-third have at least one comorbidity [58], restrictive eligibility criteria may adversely influence the representativeness of the samples to the wider colorectal cancer population. With increasing guidelines recommending participation in physical activity, including exercise, for all people with cancer, there is a clear need to ensure eligibility criteria of future studies allows for more representative samples to be investigated. Ensuring study samples reflect the diverse and complex presentations of patients in the clinical setting would require recruiting patients with characteristics such as being physically inactive, older age, poorer prognosis, presence of additional comorbidities, lower socioeconomic status and poorer access to care.

Much heterogeneity exists in the way exercise adherence and compliance is reported between studies. Approximately two-thirds of the studies (*n* = 13, 68%) reported adherence as participant attendance at exercise sessions, but did not provide data to enable assessment of exercise compliance (i.e., to what extent did participants complete prescribed exercise dosage). Therefore, while we report high adherence (86%), irrespective of intervention characteristics, compliance is unclear across all studies. Better reporting of compliance by future research will enable exploration of upper- and lower-thresholds of benefits from exercise and whether this is associated by patient characteristics, providing highly useful information for clinical practice.

Improvements in a range of health outcomes, including QoL, fatigue, aerobic fitness, depression, sleep, upper-body strength and body fat (SMD range = 0.21–0.66, *p* < 0.05) were observed. This extends findings from previous meta-analyses (limited by number of included studies and the number of outcomes these studies evaluated), which were only able to confirm benefit to physical function (SMD = 0.59, [18]), and showed no effects on fatigue (SMD = 0.18–0.21, [18, 19]) and QoL (SMD = 0.18, [18]). The findings from our subgroups analyses also support benefits can be accrued irrespective of mode, degree of supervision, timing with respect to surgery or chemotherapy, although there was some evidence that specific intervention characteristics may be particularly beneficial for specific health outcomes. For example, effect size was greater for QoL with mixed mode and supervised interventions (compared with aerobic exercise only and unsupervised interventions, respectively, Table 4). Since larger effects were observed for specific outcomes when interventions were supervised (e.g., QoL and fatigue), while unsupervised interventions appear more favourable for improvements in other outcomes (e.g., aerobic fitness, upper-body strength and reductions in body fat), prescribing targeted exercise will likely require prioritising health outcomes. This is in line with recommendations made following the most recent update to national exercise prescription guidelines for people with cancer [59]. However, it remains important to recognise that heterogeneity due to other intervention characteristics (e.g., intervention length, exercise mode and exercise intensity) may explain the differences observed in this review between supervised and unsupervised interventions.

Findings from this meta-analysis need to be considered in light of limitations. As acknowledged above, comprehensive reporting of safety and compliance data was lacking and as with many exercise trials, the included studies have the potential for recruitment bias (i.e., participants are more likely to be younger and healthier than the population from which they are drawn) and participation bias (i.e., participants are more likely to have higher exercise self-efficacy than the wider oncology population). Another limitation is that it is important to recognise that our subgroup analyses were exploratory, and lack of power may have prevented us from identifying associations that are present but not represented in our results. It is of note that most (*n* = 8, 88%) of the unsupervised interventions included in this review were short in duration (12 weeks or less). Also, it has been previously established that unsupervised interventions (*n* = 9 of 21 study arms in this review) tend to be of lower intensity than those supervised [60]. When these factors are considered together, it is possible the potential for benefit through exercise may have been underestimated and caution should be used when generalising the safety findings. Nonetheless, this systematic review and meta-analysis reflects the most comprehensive assessment of exercise and colorectal cancer studies available. Unlike previous reviews, our evaluation of safety and feasibility involved assessing all phases of treatment (pre-, during and post-treatment). Other strengths of this work included the inclusion of only RCTs, two authors assessed study quality ratings, adverse events were analysed using the Common Terminology Criteria for Adverse Event (Version 5.0), and subgroup analyses were performed to identify potential associations between disease, treatment and intervention characteristics on safety and effect outcomes.

## Conclusions

To date, this is the first systematic review and meta-analysis to: 1) undertake pooled analyses of exercise-related adverse events, and; 2) the first review article to systematically evaluate feasibility outcomes (recruitment, adherence and withdrawals) in this population. The present findings suggest that exercise following colorectal cancer diagnosis is associated with a low risk of adverse event, is feasible, and has beneficial effects on a range of health outcomes, irrespective of exercise mode, level of supervision, duration or timing with respect to chemotherapy or surgery.

## Supplementary information

**Additional file 1.**

## Data Availability

All data generated or analysed during this study are included in this published article and its supplementary information files.

## Abbreviations

CI: Confidence interval
HR: Hazard ratio
QoL: Quality of life
SD: Standard deviation
SMD: Standardized mean difference
RCT: Randomized controlled trial
RD: Risk difference

## References

[1] World Cancer Research Fund (WCRF). Colorectal cancer statistics - January 12, 2020. https://www.wcrf.org/dietandcancer/cancer-trends/colorectal-cancer-statistics. Accessed March 2020.

[2] Howlader N, Noone AM, Krapcho M, Miller D, Bishop K, Kosary CL (2016). National Cancer Institute SEER Cancer Statistics Review, 1975-2014. National Cancer Institute.

[3] Miller KD, Siegel RL, Lin CC, Mariotto AB, Kramer JL, Rowland JH (2016). Cancer treatment and survivorship statistics, 2016. CA Cancer J Clin.

[4] Center MM, Jemal A, Ward E (2009). International trends in colorectal cancer incidence rates. Cancer Epidemiol Biomark Prev.

[5] Murphy CC, Harlan LC, Lund JL, Lynch CF, Geiger AM. Patterns of Colorectal Cancer Care in the United States: 1990–2010. J Natl Cancer Inst. 2015;107(10):1–11. 10.1093/jnci/djv198 PMC4840367 26206950

[6] Bosetti C, Levi F, Rosato V, Bertuccio P, Lucchini F, Negri E (2011). Recent trends in colorectal cancer mortality in Europe. Int J Cancer.

[7] Rachet B, Maringe C, Nur U, Quaresma M, Shah A, Woods LM (2009). Population-based cancer survival trends in England and Wales up to 2007: an assessment of the NHS cancer plan for England. Lancet Oncol.

[8] Brouwer NPM, Bos ACRK, Lemmens VEPP, Tanis PJ, Hugen N, Nagtegaal ID (2018). An overview of 25 years of incidence, treatment and outcome of colorectal cancer patients. Int J Cancer.

[9] Brown SR, Mathew R, Keding A, Marshall HC, Brown JM, Jayne DG (2014). The impact of postoperative complications on long-term quality of life after curative colorectal cancer surgery. Ann Surg.

[10] Birgisson H, Pahlman L, Gunnarsson U, Glimelius B (2007). Late adverse effects of radiation therapy for rectal cancer - a systematic overview. Acta Oncol.

[11] Cheng KKF, Lee DTF (2011). Effects of pain, fatigue, insomnia, and mood disturbance on functional status and quality of life of elderly patients with cancer. Crit Rev Oncol Hematol.

[12] Vardy J, Dhillon HM, Pond GR, Rourke SB, Xu W, Dodd A (2014). Cognitive function and fatigue after diagnosis of colorectal cancer. Ann Oncol.

[13] DeCosse JJ, Cennerazzo WJ (1997). Quality-of-life management of patients with colorectal cancer. CA Cancer J Clin.

[14] Deimling GT, Sterns S, Bowman KF, Kahana B (2007). Functioning and activity participation restrictions among older adult, long-term cancer survivors. Cancer Investig.

[15] Miller KD, Nogueira L, Mariotto AB, Rowland JH, Yabroff KR, Alfano CM (2019). Cancer treatment and survivorship statistics, 2019. CA Cancer J Clin.

[16] McCabe MS, Bhatia S, Oeffinger KC, Reaman GH, Tyne C, Wollins DS (2013). American Society of Clinical Oncology statement: achieving high-quality cancer survivorship care. J Clin Oncol.

[17] Friedenreich CM, Stone CR, Cheung WY, Hayes SC. Physical activity and mortality in cancer survivors: a systematic review and meta-analysis. JNCI Cancer Spectr. 2019. 10.1093/jncics/pkz080. 10.1093/jncics/pkz080 PMC7050161 32337494

[18] Cramer H, Lauche R, Klose P, Dobos G, Langhorst J (2014). A systematic review and meta-analysis of exercise interventions for colorectal cancer patients. Eur J Cancer Care (Engl)..

[19] Brandenbarg D, Korsten JHWM, Berger MY, Berendsen AJ (2018). The effect of physical activity on fatigue among survivors of colorectal cancer: a systematic review and meta-analysis. Support care cancer.

[20] Gao R, Yu T, Liu L, Bi J, Zhao H, Tao Y, et al. Exercise intervention for post-treatment colorectal cancer survivors: a systematic review and meta-analysis. J Cancer Surviv. 2020. 10.1007/s11764-020-00900-z [Online ahead of print]. 10.1007/s11764-020-00900-z 32533468

[21] Schardt C, Adams MB, Owens T, Keitz S, Fontelo P (2007). Utilization of the PICO framework to improve searching PubMed for clinical questions. BMC Med Inform Decis Mak.

[22] Garber CE, Blissmer B, Deschenes MR, Franklin BA, Lamonte MJ, Lee IM (2011). American College of Sports Medicine position stand. Quantity and quality of exercise for developing and maintaining cardiorespiratory, musculoskeletal, and neuromotor fitness in apparently healthy adults: guidance for prescribing exercise. Med Sci Sport Exerc.

[23] Singh B, Spence RR, Steele ML, Sandler CX, Peake JM, Hayes SC. A Systematic Review and Meta-Analysis of the Safety, Feasibility, and Effect of Exercise in Women With Stage II+ Breast Cancer. Arch Phys Med Rehabil. 2018;99(12):2621–36. 10.1016/j.apmr.2018.03.026 29730319

[24] Sherrington C, Herbert RD, Maher CG, Moseley AM (2000). PEDro. A database of randomized trials and systematic reviews in physiotherapy. Man Ther.

[25] Maher CG, Sherrington C, Herbert RD, Moseley AM, Elkins M (2003). Reliability of the PEDro scale for rating quality of randomized controlled trials. Phys Ther.

[26] Armijo-Olivo S, da Costa BR, Cummings GG, Ha C, Fuentes J, Saltaji H (2015). PEDro or Cochrane to assess the quality of clinical trials? A Meta-Epidemiological Study. PLoS One.

[27] Harrison RW, Hasselblad V, Mehta RH, Levin R, Harrington RA, Alexander JH (2013). Effect of levosimendan on survival and adverse events after cardiac surgery: a meta-analysis. J Cardiothorac Vasc Anesth.

[28] Hasselblad V, Mosteller F, Littenberg B, Chalmers TC, Hunink MG, Turner JA (1995). A survey of current problems in meta-analysis. Discussion from the Agency for Health Care Policy and Research inter-PORT work group on literature review/meta-analysis. Med Care.

[29] De Jesus S, Fitzgeorge L, Unsworth K, Massel D, Suskin N, Prapavessis H (2017). Feasibility of an exercise intervention for fatigued breast cancer patients at a community-based cardiac rehabilitation program. Cancer Manag Res.

[30] Newton MJ, Hayes SC, Janda M, Webb PM, Obermair A, Eakin EG (2011). Safety, feasibility and effects of an individualised walking intervention for women undergoing chemotherapy for ovarian cancer: a pilot study. BMC Cancer.

[31] Higgins JP, Deeks JJ, Altman DG. Special topics in statistics. In: Higgins JPT, Green S, editors. Cochrane Handbook for Systematic Reviews of Interventions. Chichester: Wiley; 2008. p. 481–529.

[32] Egger M, Davey Smith G, Schneider M, Minder C (1997). Bias in meta-analysis detected by a simple, graphical test. BMJ..

[33] Thompson SG, Higgins JPT (2002). How should meta-regression analyses be undertaken and interpreted?. Stat Med.

[34] Deeks JJ, Higgins JPTAD. Analysing Data and Undertaking Meta-Analyses, Cochrane Handbook for Systematic Reviews of Interventions. In: Higgins JPT, Green S, editors. Chichester: Wiley; 2008. p. 481–529. in Cochrane Handbook for Systematic Reviews of Interventions. 2008.

[35] Cohen J (1988). Statistical power analysis for the behavioral sciences.

[36] Brown JC, Troxel AB, Ky B, Damjanov N, Zemel BS, Rickels MR (2018). Dose-response effects of aerobic exercise among Colon Cancer survivors: a randomized phase II trial. Clin Colorectal Cancer.

[37] van Waart H, Stuiver MM, van Harten WH, Geleijn E, de Maaker-Berkhof M, Schrama J (2018). Recruitment to and pilot results of the PACES randomized trial of physical exercise during adjuvant chemotherapy for colon cancer. Int J Color Dis.

[38] Bousquet-Dion G, Awasthi R, Loiselle S-E, Minnella EM, Agnihotram RV, Bergdahl A (2018). Evaluation of supervised multimodal prehabilitation programme in cancer patients undergoing colorectal resection: a randomized control trial. Acta Oncol.

[39] Cantarero-Villanueva I, Sanchez-Jimenez A, Galiano-Castillo N, Diaz-Rodriguez L, Martin-Martin L, Arroyo-Morales M (2016). Effectiveness of Lumbopelvic exercise in Colon Cancer survivors: a randomized controlled clinical trial. Med Sci Sports Exerc.

[40] Chen BP, Awasthi R, Sweet SN, Minnella EM, Bergdahl A, Santa Mina D (2017). Four-week prehabilitation program is sufficient to modify exercise behaviors and improve preoperative functional walking capacity in patients with colorectal cancer. Support Care Cancer.

[41] Courneya KS, Friedenreich CM, Quinney HA, Fields ALA, Jones LW, Fairey AS (2003). A randomized trial of exercise and quality of life in colorectal cancer survivors. Eur J Cancer Care (Engl).

[42] Gillis C, Li C, Lee L, Awasthi R, Augustin B, Gamsa A (2014). Prehabilitation versus rehabilitation: a randomized control trial in patients undergoing colorectal resection for cancer. Anesthesiology..

[43] Hubbard G, O’Carroll R, Munro J, Mutrie N, Haw S, Mason H (2016). The feasibility and acceptability of trial procedures for a pragmatic randomised controlled trial of a structured physical activity intervention for people diagnosed with colorectal cancer: findings from a pilot trial of cardiac rehabilitation versus usu. Pilot feasibility Stud.

[44] Kim DJ, Mayo NE, Carli F, Montgomery DL, Zavorsky GS (2009). Responsive measures to prehabilitation in patients undergoing bowel resection surgery. Tohoku J Exp Med.

[45] Lee MK, Kim J-Y, Kim D-I, Kang D-W, Park J-H, Ahn K-Y (2017). Effect of home-based exercise intervention on fasting insulin and Adipocytokines in colorectal cancer survivors: a randomized controlled trial. Metabolism..

[46] Van Blarigan EL, Chan H, Van Loon K, Kenfield SA, Chan JM, Mitchell E (2019). Self-monitoring and reminder text messages to increase physical activity in colorectal cancer survivors (smart pace): a pilot randomized controlled trial. BMC Cancer.

[47] Zimmer P, Trebing S, Timmers-Trebing U, Schenk A, Paust R, Bloch W (2018). Eight-week, multimodal exercise counteracts a progress of chemotherapy-induced peripheral neuropathy and improves balance and strength in metastasized colorectal cancer patients: a randomized controlled trial. Support Care Cancer.

[48] Lee MK, Kim NK, Jeon JY (2018). Effect of the 6-week home-based exercise program on physical activity level and physical fitness in colorectal cancer survivors: a randomized controlled pilot study. PLoS One.

[49] Pinto BM, Papandonatos GD, Goldstein MG, Marcus BH, Farrell N (2013). Home-based physical activity intervention for colorectal cancer survivors. Psychooncology..

[50] Cramer H, Pokhrel B, Fester C, Meier B, Gass F, Lauche R (2016). A randomized controlled bicenter trial of yoga for patients with colorectal cancer. Psychooncology..

[51] Ahn K-Y, Hur H, Kim D-H, Min J, Jeong DH, Chu SH (2013). The effects of inpatient exercise therapy on the length of hospital stay in stages I-III colon cancer patients: randomized controlled trial. Int J Color Dis.

[52] Bourke L, Thompson G, Gibson DJ, Daley A, Crank H, Adam I (2011). Pragmatic lifestyle intervention in patients recovering from colon cancer: a randomized controlled pilot study. Arch Phys Med Rehabil.

[53] Mayer DK, Landucci G, Awoyinka L, Atwood AK, Carmack CL, Demark-Wahnefried W (2018). SurvivorCHESS to increase physical activity in colon cancer survivors: can we get them moving?. J Cancer Surviv.

[54] Van Vulpen JK, Velthuis MJ, Steins Bisschop CN, Travier N, Van Den Buijs BJW, Backx FJG (2016). Effects of an exercise program in Colon Cancer patients undergoing chemotherapy. Med Sci Sports Exerc.

[55] van Rooijen SJ, Engelen MA, Scheede-Bergdahl C, Carli F, Roumen RMH, Slooter GD (2018). Systematic review of exercise training in colorectal cancer patients during treatment. Scand J Med Sci Sports.

[56] Spence R, DiSipio T, Schmitz K, Hayes S. Is unsupervised exercise following breast cancer safe for all women? Int J Phys Med Rehabil. 2014;2(197):1–8.

[57] Cancer Research UK. Bowel Cancer incidence statistics 2017 - January 13, 2010. https://www.cancerresearchuk.org/health-professional/cancer-statistics/statistics-bycancertype/bowel-cancer/incidence#ref-10. Accessed March 2020.

[58] Cuthbert CA, Hemmelgarn BR, Xu Y, Cheung WY (2018). The effect of comorbidities on outcomes in colorectal cancer survivors: a population-based cohort study. J Cancer Surviv.

[59] Hayes SC, Newton RU, Spence RR, Galvão DA (2019). The exercise and sports science Australia position statement: exercise medicine in cancer management. J Sci Med Sport.

[60] Meneses-Echavez JF, Gonzalez-Jimenez E, Ramirez-Velez R (2015). Effects of supervised exercise on cancer-related fatigue in breast cancer survivors: a systematic review and meta-analysis. BMC Cancer.

