# Supplementary material for: Exercise and colorectal cancer: a systematic review and meta-analysis of exercise safety, feasibility and effectiveness
Source: Int J Behav Nutr Phys Act. 2020 Sep 24;17:122. doi: 10.1186/s12966-020-01021-7 (PMC7513291; doi:10.1186/s12966-020-01021-7)
Supplement: Supplementary file 1 — Additional file 1. [file 12966_2020_1021_MOESM1_ESM.docx]

Supplementary content 1: Database search strategy

|  | | |
| --- | --- | --- |
|  | Medline and Pubmed | CINAHL, Cochrane, Ebscohost, ProQuest Health and Medical Complete, ProQuest Nursing and Allied Health Source, Science Direct and SPORTDiscus |
| Colorectal cancer | “Colorectal Neoplasms”[Mesh] OR  Colorectal cancer[tiab] OR  colorectal neoplasm*[tiab] OR  colon cancer[tiab] OR rectal  cancer[tiab] OR colon  neoplasm*[tiab] OR rectal  neoplasm*[tiab] OR bowel  cancer[tiab] OR cancer[tiab] OR neoplasm*[tiab] OR cancer [tiab] | (MH “Colorectal Neoplasms+”) OR (MH “Colonic  Neoplasms+”) OR TX (“colorectal cancer”) OR TX (“colon cancer”) ORTX (“rectal cancer”) |
| Physical activity | “Exercise”[Mesh] OR Exercise*[tiab]  OR physical activit*[tiab] OR  walk*[tiab] OR jog*[tiab] OR  run*[tiab] OR move*[tiab] OR  active lifestyle*[tiab] OR  sport*[tiab] OR lifestyle  intervention*[tiab] OR resistance  training [tiab] OR sedentary  behaviour [tiab] | (MH “Exercise+”) OR TX(“exercise”) OR (MH “Recovery, Exercise”) OR (MH “Aerobic Exercises+”) OR (MH  “Resistance Training”) OR (MH “Therapeutic Exercise+”)  OR (MH “Aquatic Exercises”) OR (MH “Anaerobic  Exercises”) OR (MH “Exercise Intensity”) OR (MH  “Sports Nutritional Sciences”) OR (MH “Physical  Activity”) OR (MH “Activity Therapy (Iowa NIC)”) OR  (MH “Physical Activity (Omaha)”) OR (MH“Activity and  Exercise Enhancement (Iowa NIC)+”) OR (MH “Physical  Endurance+”) OR (MH“Physical Performance”) OR (MH  “Physical Fitness+”) ORTX(“Physical activity”) OR (MH  “Lifestyle Changes”) OR (MH“Lifestyle, Sedentary”)  OR TX (“Lifestyle”) OR ((DE “Physical Activity” OR DE  “Actigraphy” OR DE “Exercise” OR DE “Physical  Fitness”) OR (DE “Exercise” OR DE “Aerobic Exercise”  OR DE “Weightlifting” OR DE “Yoga”)) OR (DE  “Activity Level”) |

Supplementary content 2: PRISMA flow diagram


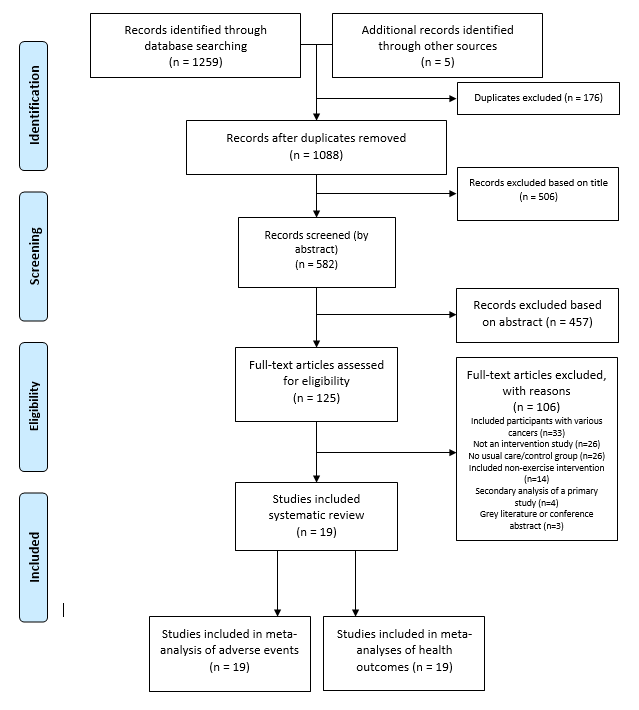


*Note: n=2 trials involved two interventions arms, therefore total of 21 intervention arms were evaluated.

Supplementary content 3: Ratings of all studies included in systematic review using the PEDro scale (n=19)

|  | PEDro Scale item number | | | | | | | | | | | |
| --- | --- | --- | --- | --- | --- | --- | --- | --- | --- | --- | --- | --- |
|  | 1 | 2 | 3 | 4 | 5 | 6 | 7 | 8 | 9 | 10 | 11 | Total score (Quality) |
| Ahn 2013 | Y | Y | N | Y | N | N | Y | Y | N | Y | Y | 6 (High) |
| Bourke 2011 | Y | Y | Y | Y | N | N | Y | Y | Y | Y | Y | 8 (High) |
| Bousquet-Dion 2018 | Y | Y | Y | Y | N | N | Y | Y | Y | Y | Y | 8 (High) |
| Brown 2017 | Y | Y | N | Y | N | N | N | Y | Y | Y | Y | 6 (High) |
| Cantarero-Villanueva 2016 | Y | Y | Y | Y | N | N | Y | Y | Y | Y | Y | 8 (High) |
| Chen 2017 | Y | Y | N | Y | N | N | N | N | N | Y | Y | 4 (Low) |
| Courneya 2003 | Y | Y | N | Y | N | N | Y | Y | Y | Y | Y | 7 (High) |
| Cramer 2016 | Y | Y | Y | Y | N | N | N | N | Y | Y | Y | 6 (High) |
| Gillis 2014 | Y | Y | Y | Y | N | N | Y | Y | Y | Y | Y | 8 (High) |
| Hubbard 2016 | Y | Y | Y | Y | N | N | N | Y | N | Y | Y | 6 (High) |
| Kim 2009 | Y | Y | N | Y | N | N | N | Y | N | Y | Y | 5 (Low) |
| Lee 2017 | Y | Y | Y | Y | N | N | N | N | Y | Y | Y | 6 (High) |
| Lee 2018 | Y | Y | Y | Y | N | N | N | N | Y | Y | Y | 6 (High) |
| Mayer 2018 | Y | Y | N | Y | N | N | N | Y | Y | Y | Y | 6 (High) |
| Pinto 2011 | Y | Y | N | Y | N | N | N | Y | Y | Y | Y | 6 (High) |
| Van Blarigan 2019 | Y | Y | Y | Y | N | N | N | Y | Y | Y | Y | 7 (High) |
| Van Vulpen 2015 | Y | Y | Y | Y | N | N | Y | Y | Y | Y | Y | 8 (High) |
| van Waart 2018 | Y | Y | Y | Y | N | N | N | Y | Y | Y | Y | 8 (High) |
| Zimmer 2018 | Y | Y | Y | Y | N | N | N | N | Y | Y | Y | 6 (High) |
| Pedro scale items: 1. Eligibility criteria; 2. Subjects randomly allocated; 4. Groups similar at baseline; 5. Subject blinding; 6. Therapist blinding; 7. Assessor blinding; 8. Outcome obtained from >85% of subjects; 9. Intention to treat; 10. Results of between-group comparisons; 11. Point and variability measures.  A score 6 or higher was considered high quality and trials receiving less than 6 were classified as low quality. | | | | | | | | | | | | |

Supplementary content 4. Overview of all reasons for withdrawal, separated as health or non-health-related reasons (n=19).

|  | Withdrawals from intervention group  n= (89 withdrawals out of total 670 participants) | | Withdrawals from usual care group  n= (85 withdrawals out of 623 participants) | |
| --- | --- | --- | --- | --- |
|  | <12-week interventions n=37 | >12-week interventions n=52 | <12-week interventions n=37 | >12-week interventions n=48 |
| Reason for withdrawals | **Health-related reasons n=:** 8   - Required neoadjuvant therapy due to metastatic disease n=2 - Acute illness n=1 - Fatigue & malaise n=2 - Pneumonia n=1 - Emergency surgery required n=1 - Death n=1   **Non-health-related reasons or other n=29:**   - No reason for withdrawal or reason not reported n=11 - Surgery was not required or did not have surgery n=5 - Uncontactable or lost to follow-up n=4 Family reasons n=1 - Scheduling problems n=3 - Not assessed at surgery n=3 - Never started n=1 - Too busy n=1 | **Health-related reasons n=10:**   - Unspecified medical complication n=5 - Stroke n=1 - Diagnosed with lung cancer n=1 - Death (due to perforated cholecystitis) n=1 - Cancer recurrence n=1 - Metastasis n=1   **Non-health-related reasons or other n=42:**   - No reason for withdrawal or reason not reported n=21 - Uncontactable or lost to follow-up n=19 - Did not want to do moderate exercise, n=1 - Personal reasons n=1 | **Health-related reasons n=8:**   - Required neoadjuvant therapy due to metastatic disease n=2 - Acute illness n=1 - Unspecified health problems n=1 - Progressive disease with cough n=1 - Depression n=1 - Psychological reasons n=1 - Death due to sepsis n=1   **Non-health-related reasons or other n=29:**   - No reason for withdrawal or reason not reported n=13 - Surgery was not required n=4 - Uncontactable or lost to follow-up n=4 - Too busy n=2 - Operated at another hospital n=2 - Not assessed at surgery n=2 - Personal reasons n=1 - Family reasons n=1 | **Health-related reasons n=8:**   - Unspecified medical reasons n=4 - Cancer recurrence n=1 - Psychological reasons n=1 - Death n=1   **Non-health-related reasons or other n=40:**   - Uncontactable or lost to follow-up n=21 - No reason for withdrawal or reason not reported n=16 - No time n=1 - Withdrew due to obtaining own Fitbit during the study period n=1 - Disappointment of group allocation n=1 |

Supplementary content 5. Overview of all instruments and methods used to assess all outcomes of interest.

| Quality of life | - Functional Assessment of Cancer Therapy-Colorectal (FACT-C) (n=7) - European Organization for Research and Treatment (EORTC QLQ-C30 version 3) (n=3) - European Quality of life 5 Dimensions (EQ-5D) (n=1) - Gynecologic Oncology Group Neurotoxicity (n=1) |
| --- | --- |
| Aerobic fitness | - Six-minute walk test (n=7) - Step test (n=1) - 150-second isometric fatigue protocol at 20% of MVT (n=1) - Modified Balke Treadmill Test (n=1) - Cycling ergometer test to volitional fatigue (VO2max test) (n=2) - Steep ramp test (n=1) - Submaximal treadmill walk test (n=1) |
| Fatigue | - Functional Assessment of Cancer Therapy-Fatigue (FACT-F) (n=5) - Fatigue Symptom Inventory (n=1) - Abbreviated Fatigue Questionnaire (n=1) - Multidimensional Fatigue Inventory (n=2) |
| Upper-body strength | - Push-up test (n=2) - Hand-held dynamometer (n=1) - One-repetition maximum test (n=1) |
| Lower-body strength | - Sit-to-stand test (n=4) - Isokinetic dynamometry (n=1) - Hand-held dynamometer (n=1) - One-repetition maximum test (n=1) |
| Anxiety | - Hospital Anxiety and Depression Scale (n=4) - State-Trait Anxiety Inventory (n=1) |
| Depression | - Hospital Anxiety and Depression Scale (n=4) - Centre for Epidemiological Studies Depression scale (n=1) |
| Sleep | - Pittsburgh Sleep Quality Inventory (n=2) |
| Body fat | - Bioimpedance spectroscopy (n=4) - Skinfold measurement (n=1) - Dual-energy X-ray absorptiometry (n=1) |
| Body mass index | n=5 |
